# Multi-Step Natural Gas Load Forecasting Incorporating Data Complexity Analysis with Finite Features

**DOI:** 10.3390/e27070671

**Published:** 2025-06-23

**Authors:** Ning Tian, Bilin Shao, Huibin Zeng, Meng Ren, Wei Zhao, Xue Zhao, Shuqiang Wu

**Affiliations:** 1 School of Management, Xi’an University of Architecture and Technology, Xi’an 710055, China; tning4891@xauat.edu.cn (N.T.);; 2 School of Economics and Management, Chongqing Normal University, Chongqing 401331, China; zenghuibin@xauat.edu.cn; 3 School of Mechanical and Electrical Engineering, Shaanxi University of Science and Technology, Xi’an 710021, China

**Keywords:** load forecasting, natural gas loadings, complexity feature, ensemble learning, data decomposition, deep learning

## Abstract

Data complexity directly affects the dynamics of complex systems, which in turn influences the accuracy and robustness of forecasting models. However, the load data exhibit complex features such as self-similarity, long-term memory, randomness, and chaos. This study aims to quantify and evaluate the complexity features of natural gas loads and to develop a multi-step-ahead forecasting model that integrates data decomposition and ensemble deep learning while considering these complexity features. Firstly, the complexity features of the series are quantified by rolling the fractal dimension, Hurst exponent, sample entropy, and maximum Lyapunov exponent. The analysis contributes to understanding data characteristics and provides information on complex features. Secondly, the ensemble learning eXtreme Gradient Boosting (XGBoost) can effectively screen complexity features and meteorological factors. Concurrently, variational mode decomposition (VMD) provides frequency-domain decomposition capability, while the gated recurrent unit (GRU) captures long-term dependencies. This synergy enables effective learning of local features and long-term temporal patterns, resulting in precise predictions. The results indicate that compared to other models, the proposed method (XGBoost-VMD-GRU considering complex features) demonstrates superior performance in forecasting, with R^2^ of 0.9922, 0.9860, and 0.9679 for one-step, three-step, and six-step prediction, respectively. This study aims to bring innovative ideas to load forecasting by integrating complex features into the decomposition forecasting framework.

## 1. Introduction

At present, the global energy emission reduction task is very urgent; the total global energy carbon emissions increased by 2%, exceeding 40 billion tons of carbon dioxide for the first time [1]. As an important means to mitigate climate change and reduce carbon emissions, the research and application of clean energy can promote sustainable economic development and enhance the security and stability of energy systems. In the energy transition, new energy sources, such as wind and solar energy, have the characteristics of instability, while natural gas has become an ideal transition energy to reduce carbon emissions and achieve net-zero emissions [2]. Natural gas can provide a reliable supplement to clean energy and make up for the instability of new energy sources. Its low carbon emissions, efficient combustion, flexible transportation modes, and abundant reserves make it play an important role in the energy transition [3]. According to the 73rd edition of the Statistical Yearbook of World Energy, global primary energy consumption reached 620 EJ in 2023, with fossil energy sources still dominating, accounting for 81.5% of the global primary energy structure. Although global natural gas demand remained relatively stable, with an increase of only 0.02%—possibly due to pandemic-related policies—natural gas consumption exhibited a steady growth trend until 2022. However, natural gas still plays an important role in the global energy structure as a less carbon-intensive fossil fuel [1]. Energy issues span from macro to micro levels, impacting national economic sustainability and the stability of people’s livelihoods. Accurately forecasting natural gas load (NGL) can optimize energy utilization, enhance supply security, reduce production costs, facilitate the formulation of sound energy policies, and promote economic development. Simultaneously, for industries and enterprises, forecasting aids in adjusting production and supply strategies to maintain market supply-demand equilibrium.

NGL forecasting models typically encompass statistical models, machine learning (ML) models, grey models, fuzzy logic models, and their combinations. Statistical models and ML models are widely used for short- to medium-term forecasting, leveraging their capacity to manage intricate market dynamics and fluctuations in supply and demand effectively [4]. Statistical models offer advantages in prediction tasks due to their rapid computational speed and ability to capture temporal trends [5,6]. However, they exhibit relatively weaker predictive capabilities when faced with complex data or nonlinear relationships [7]. In contrast, ML models can handle nonlinear and volatile data effectively, possessing strong generalization abilities and robustness against noise. Wei et al. utilized support vector regression (SVR) for short-term natural gas consumption (NGC) forecasting and optimized the model’s hyperparameters using a genetic algorithm (GA) [8]. Rehman et al. compared the performance of stepwise multiple linear regression, multilayer perceptron, and long short-term memory (LSTM) models in hourly natural gas demand forecasting. They emphasized LSTM’s advantages in predictive accuracy and management of extreme events [9]. Wang et al. applied the LMDI method to analyze influencing factors and developed a PSO-LSTM model to forecast natural gas consumption in China. The model demonstrated high prediction accuracy, and the results indicate a continued increase in future energy demand [10].

Deep learning techniques demonstrate powerful predictive capabilities in time series forecasting. However, when confronted with complex, highly volatile, and stochastic data, individual prediction algorithms often prove inadequate, and other techniques are needed to assist prediction to address these challenges effectively. Decomposition algorithms emerged from the need for complex data structures. The original load data are decomposed into simpler, more interpretable components. This facilitates more accurate identification of underlying patterns and trends. Ding et al. introduced the Dual Convolution with a Seasonal Decomposition Network, employing multiple seasonal decompositions using loss to address common seasonal variations and periodic fluctuations in NGC data. Experimental results indicate significant potential for the practical application of this approach [11]. Peng et al. combined local mean decomposition, wavelet threshold denoising, and LSTM in various configurations, discussing their application in NGC forecasting [12]. Jiang et al. utilized variational mode decomposition (VMD), wavelet packet decomposition, and LSTM to forecast complex nonlinear natural gas data. The model exhibited outstanding performance in experiments [13]. Wu and Wang significantly improved the quality of raw data preprocessing by using a two-stage decomposition strategy that combines EMD and EEMD. This approach further enhanced the extraction of valuable predictive information [14]. Zhao et al. proposed a deep learning model based on VMD-CNN-LSTM with a self-attention mechanism for natural gas load interval forecasting. They also developed a hierarchical early warning system to enhance prediction-based risk management [15].

In natural gas demand forecasting, it is crucial to consider the influence of external factors on prediction outcomes. Common short-term influencing factors include temperature, holidays, seasons, and others, which can significantly influence consumption patterns and production demands of natural gas. However, an excess of external factors may lead to increased redundancy in the model, thereby reducing the accuracy and interpretability of predictions. Therefore, effective feature selection is necessary. The most common method for feature selection is correlation analysis [16,17]. However, its effectiveness in complex real-world scenarios is often less than satisfactory. In contrast, feature selection based on embedding methods integrates the feature selection process into model training. By ranking the importance of features, it can effectively identify critical features [18]. Rao et al. employed random forest analysis to assess the importance of factors influencing China’s energy demand, effectively addressing data redundancy and multicollinearity, thereby offering a reliable method for energy demand forecasting [19]. Zeng et al. integrated the CatBoost algorithm into a hybrid prediction framework, significantly enhancing predictive performance by synthesizing the fluctuation parameters of GARCH models and the importance of meteorological conditions and other influencing factors [20].

With the advancement of data science and machine learning technologies, integrating complex features for NGL forecasting has emerged as a new research direction. Current research in natural gas forecasting primarily focuses on combining different models, with data processing limited to basic operations such as preprocessing and feature analysis of raw data. However, due to the inherent complexity and variability of data, relying solely on collected influencing factors may fail to capture all the information necessary for accurate load forecasting. Therefore, achieving more accurate predictions necessitates a deeper focus on the relationship between the complexity and predictability of time series data [21]. Self-similarity can characterize the analogous patterns of load data across various temporal scales. Randomness describes the degree of irregularity present in the load data. Karaca et al. integrated fractal analysis and entropy analysis in their predictive experiments using stock index datasets, achieving higher accuracy in stock data forecasts. This approach provides a representation of self-similarity and complexity for stock prediction [22]. Similarly, Kim et al. combined effective transfer entropy with machine learning algorithms to enhance the prediction of stock prices [23]. Long-term memory describes the persistent influence of past values on future values within load data. Chaos is utilized to assess the degree of chaotic behavior within a system, capturing the unpredictability of the data. Raubitzek and Neubauer found that the integration of complexity analysis results, such as approximate entropy, sample entropy, and fractal dimension, with machine learning outcomes significantly enhances the identification of predictable periods. They also explored the incorporation of complexity analysis from chaos theory to improve the accuracy of machine learning predictions [21]. In the field of natural gas, Bai et al. proposed a complexity measurement method that integrates coefficient of variation analysis, local fluctuation coefficient analysis, and kurtosis analysis. This experiment utilizes the comprehensive complexity of data to assess the impact of various factors on prediction accuracy [24]. Wei et al. employed the analytic hierarchy process to comprehensively address irregularities, complex cyclical variations, and volatility in consumption data. They established complexity measurement indicators to assess the relationship between complexity and predictive performance [25]. However, the above studies have not directly integrated specific complexity features of the data into the forecasting framework, thereby limiting their practical contributions to enhancing predictive performance. They have mainly focused on assessing the correlation between data of varying complexities and predictive performance, while a detailed exploration of the specific data characteristics of complexity features remains to be thoroughly investigated.

In summary, current research lacks a thorough exploration of how to organically integrate complexity features into forecasting frameworks. A thorough exploration and precise quantification of intrinsic complexity characteristics, such as self-similarity, long-term memory, randomness, and chaos in data, are still lacking. Considering these complex features comprehensively becomes a crucial factor in enhancing predictive accuracy in the study. Although NGL forecasting techniques have become increasingly mature, their predictive performance often remains unsatisfactory when handling data with varying levels of complexity. Moreover, these models tend to underperform in capturing long-term dependencies and volatile patterns, posing a critical challenge that requires further investigation.

To achieve more precise predictions, it is essential to delve into the complex features of historical sequences and their impact on predictive performance. By integrating embedding-based feature engineering with load decomposition techniques, predictive models effectively identify inherent data patterns and dynamic trends. This framework significantly improves forecasting accuracy and robustness. In this work, an innovative load-forecasting model that takes into account the characteristics of complexity is proposed. The main contributions of this study are as follows:

(1) This study utilized fractal dimension, Hurst exponent, sample entropy, and maximum Lyapunov exponent to analyze the self-similarity, long-term memory, randomness, and chaotic characteristics of historical NGL data, thereby delving into the complexity of time series. These analytical findings not only contribute to a comprehensive understanding of data properties but also furnish crucial complexity feature information for predictive models.
(2) This study represents the first attempt to organically integrate complexity features into a load-forecasting model, establishing a multi-step prediction model for daily NGC. By integrating GRU with XGBoost and VMD, significant improvements in prediction accuracy and robustness were achieved. Feature importance ranking using XGBoost confirms the significance of complexity features and certain meteorological factors in forecasting. In practical applications, multi-step preprocessing and prediction strategies are designed for complex long-term prediction needs.
(3) This study rigorously verifies the effectiveness and feasibility of the proposed method using detailed NGL data collected from real-world sites spanning from 2016 to 2022. Additionally, to assess its robustness in practical applications, extensive simulation experiments were conducted by introducing various noise disturbances with different distributions into the original data. These experiments confirmed the superior performance of the method in real-world scenarios and demonstrated its strong adaptability to complex data and noise interference.

The rest of the paper is organized as follows. Section 2 describes the complexity features and the related methods of the proposed model. Section 3 elaborates on the main structure of the proposed hybrid model. Section 4 provides a detailed description of the experiments, focusing on feature importance, data decomposition, complexity feature analysis, and comparative result analysis. Finally, Section 5 summarizes the conclusions and proposes future directions.

## 2. Methods

### 2.1. Analysis of Data Complexity

#### 2.1.1. Fractal Dimension

The fractal dimension (FD) is a geometric metric used to quantify the complexity of an object. It is commonly used to characterize the geometric properties of both natural and artificial complex structures. The FD method in time series data primarily characterizes the complexity of signals [26]. FD analyzes the internal structure of time series data within the time domain without requiring phase space reconstruction. Its objective is to reveal self-similarity and long-term dependencies in data, thereby further understanding the dynamic characteristics and behavioral patterns of the data.

This study employs the method proposed by Higuchi to compute the FD. The principle is analogous to fractal geometry [27], where complex geometric structures are decomposed into simpler substructures, and their complexity is described through scale transformations. In one-dimensional time series analysis, the FD is estimated by assessing the relationship between the scale length and the corresponding curve length across multiple resolutions. The FD is calculated by averaging these measurements, providing a quantitative description of the complexity of the time series. The underlying principle is as follows.

Step 1: Split the original sequence data in different degrees to form a new subsequence, $X_{k}^{m}$.

(1) $$X_{k}^{m}=\left[X(m),X(m+k),X(m+2k),\ldots ,X\left(m+\left[\frac{\left(N-m\right)}{k}\right]k\right)\right]$$

where *m* refers to the initial time point, m=1, 2, …, k; *k* is the delay interval; and *N* is the length of the time series.

Step 2: Compute the curve length, $L_{m}\left(k\right)$, of the subsequence $X_{k}^{m}$.

(2) $$L_{m}(k)=\frac{1}{k}\left(\sum_{i=1}^{\left[\frac{N-m}{k}\right]}\left|X(m+ik)-X(m+(i-1)k)\right|\right)\left(\frac{N-1}{[\frac{N-m}{k}]k}\right)$$

Step 3: Compute the average $L\left(k\right)$ by averaging across different *m* values for the same k.

(3) $$L(k)=\frac{1}{k}\;\sum_{m=1}^{k}L_{m}(k)\;$$

Step 4: Fit different values of *k* in a logarithmic coordinate system, where the slope represents the *FD*.

(4) $$FD=\frac{\ln(L(k))}{\ln(\frac{1}{k})}$$

#### 2.1.2. Hurst Exponent

At different time scales, the long-term correlations of sequences vary. To assess the long-term memory of time series, this study employs rescaled range analysis (R/S analysis) to calculate the Hurst exponent (HE) of the time series [28].

The R/S measure is a classical and effective method used to assess long-term correlations by comparing the variations of time series across different scales, facilitating a deeper understanding of the underlying trends in time series data. The principle behind it is as follows.

Step 1: Divide the original sequence into i non-overlapping subseries $X_{ij}$ of length j.

Step 2: Compute the mean of each subgroup, $\bar{X_{a}}$, where a=1, 2,…, i, and b=1, 2,…, j.

(5) $$\bar{X_{a}}=\frac{1}{j}\;\sum_{b=1}^{j}X_{ab}$$

Step 3: Within subsequence a, sequentially calculate the cumulative deviations $Y_{ab}$ for the first b points of the subseries.

(6) $$Y_{ab}=\sum_{k=1}^{b}\left(X_{ak}-\bar{X_{a}}\right)$$

Step 4: Calculate the range $R_{a}$ of subsequence a, representing the fluctuation amplitude of the sequence.

(7) $$R_{a}=\max(Y_{ab})-\min(Y_{ab})$$

Step 5: Compute the standard deviation $S_{a}$ of subsequence a.

(8) $$S_{a}=\sqrt{\frac{\sum_{b=1}^{j}{\left(X_{ab}-\bar{X_{a}}\right)}^{2}}{j-1}}$$

Step 6: Standardize the range by using the standard deviation to obtain the re-scaled sequence ${RS}_{a}=\frac{R_{a}}{S_{a}}$. Then compute the average ${\left(R/S\right)}_{j}$ of ${RS}_{a}$ for each subsequence. Repeat the above steps for subsequences of different lengths to obtain ${\left(R/S\right)}_{j}$ at different lengths j.

Step 7: Calculate the slope of the fitting curve $log({\left(\frac{R}{S}\right)}_{j})\sim log(j)$ to obtain HE.

#### 2.1.3. Sample Entropy

Entropy quantifies the disorder level within a system. Higher entropy values correspond to increased disorder, consequently reducing the predictability of system states. Sample entropy (SE) is a computational method in information theory that improves upon approximate entropy calculations. Richman and Moorman introduced this method for measuring time series complexity, aiming to address the limitations of approximate entropy that are dependent on data length [29]. In NGL prediction, the complexity of load data is characterized by computing SE, which further reflects the predictability of the data, describing its randomness and uncertainty. The underlying principle is as follows.

Step 1: For a time series of n elements $\left[x_{1},x_{2},\ldots ,x_{n}\right]$, generate a set of vector sequences $X_{m}\left(i\right)\;$*=* ($x_{i},x_{i+1},\ldots ,\;x_{i+m-1}$) with subset dimension *m*, where i = 1, 2, …, n−m.

Step 2: Define a distance function $d[X_{m}\left(i\right),X_{m}\left(j\right)]$, where j≠i.

(9) $$d[X_{m}(i),X_{m}(j)]=\underset{k=0,1,2,\ldots ,m-1}{\max}\left[\left|X(i+k)-X(j+k)\right|\right]$$

Step 3: Count the number of indices j∈(1, n−m+1) for which the distance between $X_{m}\left(i\right)\;$ and $X_{m}\left(j\right)\;$ is less than or equal to *r*, denoted as the approximate count $\psi_{i}^{m,r}$. Then, calculate the approximate ratio $\varphi_{i}^{m,r}$ and define $\phi_{i}^{m,r}$ as the probability of matching *m* points between two sequences under a similarity threshold *r*.

(10) $$\varphi_{i}^{m,r}=\frac{1}{n-m+1}\psi_{i}^{m,r}$$

(11) $$\phi^{m,r}=\frac{1}{n-m}\sum_{i=1}^{n-m}\varphi_{i}^{m,r}$$

Step 4: Increase the dimension to m+1 and repeat the aforementioned process to obtain $\phi^{m+1,r}$.

(12) $$\phi^{m+1,r}=\frac{1}{n-m}\sum_{i=1}^{n-m}\varphi_{i}^{m+1,r}$$

Step 5: Calculate the *SE*.

(13) $$SE=-\ln(\frac{\phi^{m+1,r}}{\phi^{m,r}})$$

#### 2.1.4. Maximum Lyapunov Exponent

The Maximum Lyapunov Exponent (MLE), introduced by Lyapunov [30], quantifies the exponential growth rate of nearby trajectories in a system. It characterizes the system’s sensitivity to initial conditions and serves as a measure of predictability in time series [31]. A larger value of the Lyapunov exponent indicates greater chaos in the time series and, correspondingly, lower predictability. This study exclusively computes MLE because it represents the maximum systemic chaos and sensitivity of sequences [32]. The commonly used algorithms for this purpose are the Wolf algorithm [33] and the Rosenstein algorithm [34]. A positive MLE indicates that the system exhibits hyperchaos [35,36], characterized by significant disorder and complexity. A negative value of MLE means that the system is stable. Since the research object of this paper is a set of NGL data, it is more robust to use Rosenstein’s algorithm to calculate MLE for longer time series. The specific calculation formula is as follows.

Step 1: For a given sequence data $x_{i}=[x_{1},x_{2},\ldots x_{n}]$, reconstruct the sequence points as $X_{m}^{\tau }(i)$ using attractors.

(14) $$X_{m}^{\tau }(i)=[x_{i},x_{i+\tau },\ldots ,x_{\left(i+(m-1)\tau \right)}]$$

Step 2: Calculate the distance $d_{i}\left(0\right)$ between two neighboring points on the trajectory.

(15) $$d_{i}(0)=\min_{X_{m}^{\tau }(i)}d(X_{m}^{\tau }(j),X_{m}^{\tau }(i))$$

Step 3: When identifying nearest neighbors, the constraint requires that the time interval between them should exceed the average period of the time series. Here, λ represents the MLE, while $C_{j}$ denotes the initial interval.

(16) $$d_{j}(i)\approx C_{j}e^{\lambda (i\Delta t)}$$

Step 4: Take the logarithm of both sides of the above equation.

(17) $$\ln(d_{j}(i))\approx \ln C_{j}+\lambda (i\Delta t)$$

Step 5: The above equation represents a set of similarly parallel lines with slopes proportional to λ. Estimate the MLE by fitting the average line using the least squares method, where <> denotes the average of all *j* points.

(18) $$y(i)=\frac{1}{\Delta t}\langle \ln(d_{j}(i))\rangle$$

In summary, Table 1 comprehensively outlines the four complexity analysis metrics, detailing their theoretical definitions, typical value ranges, and interpretive significance for practical applications. The listed typical value ranges represent the commonly observed behavior of the four complexity analysis metrics in time series applications. They should not be regarded as their complete value domains under broader mathematical definitions.

### 2.2. Extreme Gradient Boosting

From an ensemble learning perspective, eXtreme Gradient Boosting (XGBoost) utilizes a combination strategy of multiple base learners, continuously optimizing the overall performance of the model through weighted strategies in each iteration [37]. Compared to other tree models, XGBoost demonstrates greater flexibility in feature engineering and it incorporates regularization techniques to enhance model generalization capabilities. This study utilizes XGBoost to rank feature importance, thereby effectively providing critical input features for subsequent prediction methods. During the process of tree splitting, the calculation of Gain is shown in Equation (19):

(19) $$Gain=\frac{1}{2}\left[\frac{G_{L}^{2}}{H_{L}+\lambda }+\frac{G_{R}^{2}}{H_{R}+\lambda }-\frac{{\left(G_{L}+G_{R}\right)}^{2}}{H_{L}+H_{R}+\lambda }\right]-\gamma$$

Here, $G_{L}$ and $G_{R}$ denote the sum of first-order derivatives of the loss function for the left and right nodes, respectively. $H_{L}$ and $H_{R}$ denote the sum of second-order derivatives of the loss function for the left and right nodes, respectively. Specifically, *j* represents the node and *i* represents the sample of each node, $G_{j}=\sum_{\left(i\in I_{j}\right)}g_{i}$, $H_{j}=\sum_{\left(i\in I_{j}\right)}h_{i}$. $g_{i}$ represents the first-order derivative of the loss function, and $h_{i}$ represents the second-order derivative of the loss function. λ and γ are regularization parameters.

### 2.3. Variational Mode Decomposition

Short-term load data are inherently complex due to their diverse cyclic patterns, underlying trends, and the presence of noise. Therefore, accurate prediction requires careful analysis to effectively capture the relationships among these features. This paper introduces the variational mode decomposition (VMD) technique to handle complex NGL data, which effectively captures trend information within the data and mitigates noise interference on prediction outcomes. Moreover, the VMD offers controllable mode decomposition and flexible parameter adjustment capabilities, allowing adaptation to specific requirements. Furthermore, it demonstrates relatively high computational efficiency [38]. The detailed principles are outlined as follows.

The VMD transforms the decomposition process into an optimization problem, expressed through the following constraints:

(20) $$\left\{\begin{array}{c} \underset{\left\{u_{k}\},\{\omega_{k}\right\}}{\min}\left\{{\sum_{k=1}^{k}\left\|\partial_{t}[(\delta (t)+\frac{j}{\pi t})u_{k}(t)]e^{-jw_{k}t}\right\|}_{2}^{2}\right\}\\ \sum u_{k}=f(t) \end{array}\right.$$

in which $\left\{u_{k}\right\}=\{u_{1},u_{2}\ldots u_{k}\}$ represents the *k* decomposed mode components; $\left\{\omega_{k}\right\}=\{\omega_{1},\omega_{2}\ldots \omega_{k}\}$ denotes the central frequencies of each mode; δ(t) is the Dirac distribution; the operator $e^{-jw_{k}t}$ can shift the central frequency of the mode component to the baseband; and $\sum u_{k}=f(t)$ indicates the sum of all mode components is equal to the original signal.

Introducing the quadratic penalty factor, *α*, and the Lagrange penalty operator, σ(t), improves the solution of the aforementioned variational constraints optimally. The expanded Lagrange expression is given by the following equation:

(21) $$\begin{array}{l} L(\{u_{k}(t)\},\{\omega_{k}(t)\},\sigma (t))=\alpha {\sum_{k=1}^{k}\left\|\partial_{t}[(\delta (t)+\frac{j}{\pi t})u_{k}(t)]e^{-jw_{k}t}\right\|}_{2}^{2}\\ +{\left\|f(t)-\sum_{k=1}^{k}u_{k}(t)\right\|}_{2}^{2}+[\sigma (t),f(t)-\sum_{k=1}^{k}u_{k}(t)] \end{array}$$

Applying the alternating direction method of multipliers to address the variational problem described above involves iteratively updating $u_{k}^{n+1}(t)$, $\omega_{k}^{n+1}\left(t\right)$, and $\sigma_{k}^{n+1}(t)$. This approach ultimately yields the saddle point of the unconstrained model, following these steps:

Step 1: Initialize ${\tilde{u}}_{k}^{1},{\tilde{\omega }}_{k}^{1},{\tilde{\sigma }}_{k}^{1}$, and *n*.

Step2: Increment n=n+1 and k=k+1 iteratively until the specified number of layers is reached, terminating the loop. Update formulas for mode components and central frequencies as follows:

(22) $${\tilde{u}}_{k}^{\left(n+1\right)}=\frac{\left\{\tilde{f}(\omega )-\sum_{i\leq k}{\tilde{u}}_{i}^{n+1}(\omega )-\sum_{i\geq k}{\tilde{u}}_{i}^{n}(\omega )+\frac{{\tilde{\sigma }}^{n}(\omega )}{2}\right\}}{\left(1+2\alpha (\omega -\omega_{k}^{n}{)}^{2}\right)}$$

where ${\tilde{u}}_{k}^{n+1}$, $\tilde{f}\left(\omega \right)$, and ${\tilde{\sigma }}^{n}\left(\omega \right),$ respectively, denote the Fourier transforms of $u_{k}^{n}(t)$, $f\left(t\right)\;$, and $\sigma_{k}^{n}(t)$; and ${\tilde{u}}_{i}^{n+1}\left(\omega \right)$ represents the result of $\tilde{f}\left(\omega \right)-\sum_{i\leq k}{\tilde{u}}_{i}t\left(\omega \right)$ passed through a wiener filter. The algorithm re-estimates central frequencies based on the power spectra of each component.

(23) $$\omega_{k+1}^{n}=\frac{\int_{0}^{\infty }\omega {\left|{\tilde{u}}_{k}^{n+1}(\omega )\right|}^{2}}{\int_{0}^{\infty }{\left|{\tilde{u}}_{k}^{n+1}(\omega )\right|}^{2}}$$

Step 3: Update σ with a precision threshold ξ; terminate the iteration when $\frac{\sum_{k}{\left\|{\tilde{u}}_{k}^{n+1}-{\tilde{u}}_{k}^{n}\right\|}_{2}^{2}}{{\left\|{\tilde{u}}_{k}^{n}\right\|}_{2}^{2}}\leq \xi$.

(24) $${\tilde{\sigma }}^{n+1}\leftarrow {\tilde{\sigma }}^{n+1}(\omega )+\tau (\tilde{f}(\omega )-{\tilde{u}}_{k}^{n+1}(\omega ))$$

### 2.4. Gated Recurrent Unit

**Figure 1:**
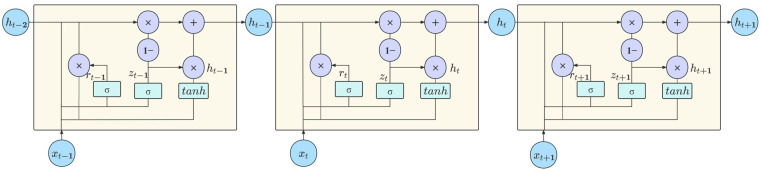
Structure diagram of GRU.

The gated recurrent unit (GRU) is a deep learning model for sequence modeling and prediction originally proposed by [39]. LSTM addresses the gradient vanishing problem of RNNs by employing gated units designed to capture long-term dependencies. However, LSTM incorporates input gates, forget gates, and output gates, rendering its structure more complex. The GRU further simplifies the internal model structure based on LSTM, including the update gate and the reset gate. The GRU utilizes an update gate to regulate how much information from the previous unit should be retained, while the reset gate controls whether the stored information from the preceding unit should be combined with the input to the next unit. The design of GRU aims to simplify the gating mechanism while enhancing computational efficiency without compromising performance. The specific structure is illustrated in Figure 1.

The GRU neuron comprises a reset gate $r_{t}$, an update gate $z_{t}$, a candidate hidden state ${\tilde{h}}_{t}$, and the final hidden state $h_{t}$, where t denotes the current time step. Detailed expressions are given by Equations (25)–(28), in which *W* denotes the weight matrix, b represents the bias term, σ denotes the sigmoid activation function, tanh denotes the hyperbolic tangent activation function, and ⊙ represents the Hadamard product.

(25) $$r_{t}=\sigma (W_{r}[h_{t-1},x_{t}]+b_{r})$$

(26) $$z_{t}=\sigma (W_{z}[h_{t-1},x_{t}]+b_{z})$$

(27) $${\tilde{h}}_{t}=\tanh(W_{h}[r_{t}⊙h_{t-1},x_{t}]+b_{h})$$

(28) $$h_{t}=(1-z_{t})⊙h_{t-1}+z_{t}⊙{\tilde{h}}_{t}$$

The reset gate $r_{t}$, determined by the sigmoid function, regulates how much information from the previous unit’s hidden state $h_{t-1}$ flows into the candidate hidden state ${\tilde{h}}_{t}$. It controls how the hidden state from the previous timestep influences the candidate’s hidden state at the current timestep. The update gate $z_{t}$, determined by the sigmoid function, regulates how much information from the input variable $x_{t}$ and the previous time step’s hidden state $h_{t-1}$ output flows into the current hidden state $h_{t}$. The ${\tilde{h}}_{t}$ is the candidate hidden state updated based on $r_{t}$, incorporating information from the reset gate and the current input. Based on $r_{t}$ and the previous timestep’s hidden state $h_{t-1}$, computed using the Hadamard product, the resulting vector is added to the current timestep input $x_{t}$. This combined result undergoes a tanh activation function to yield ${\tilde{h}}_{t}$. The final hidden state $h_{t}$, determined by the update gate, combines useful information from the current candidate state ${\tilde{h}}_{t}$ and the previous state $h_{t-1}$. It is a weighted combination resulting from the update gate. It is worth mentioning that the activation function can also be other functions such as rectified linear unit (ReLU) or scaled exponential linear unit (SELU). The sigmoid and tanh functions are generally used.

## 3. Main Structure of the Proposed Hybrid Model

**Figure 2:**
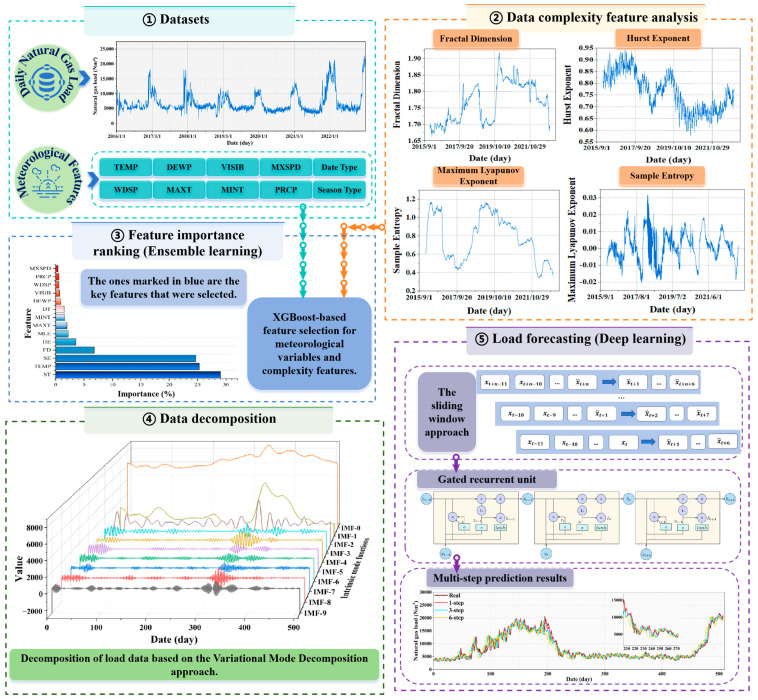
A load-forecasting framework based on data decomposition and ensemble deep learning for incorporating complex features.

The complexity of historical NGL data requires a deeper understanding of its features of the historical series and their impact on forecasting to achieve more accurate forecasts. This paper innovatively proposes a load-forecasting model based on data decomposition and ensemble deep learning by incorporating complex features. This approach establishes a comprehensive and efficient forecasting framework that not only enhances prediction accuracy but also strengthens the model’s adaptability and robustness to future variations. The process of the hybrid forecasting framework is illustrated in Figure 2, providing a clear depiction of the entire workflow from data collection to final prediction. This ensures systematic and methodological rigor in NGL forecasting.

The proposed framework includes five core components: ① Data collection involves obtaining raw data from multiple sources to ensure comprehensiveness and accuracy. ② This study constructs complexity features that capture NGL’s self-similarity, long-term memory, randomness, and chaos to augment the predictive model’s performance. ③ The contribution of meteorological and complexity features to the forecasting model is assessed using ensemble learning with XGBoost technology. ④ Since direct modeling on highly complex data often leads to suboptimal results, the VMD method is used to decompose historical load data into several intrinsic mode functions (IMFs). ⑤ Finally, the selected key features are combined with the decomposed subcomponents as inputs to the GRU forecasting model to collectively perform the multi-step prediction task.

To meet the requirements of real-world applications, this study conducted multi-step forecasting of NGL by utilizing a multi-feature dataset and comparative modeling experiments. The results provide decision-making support for developing sustainable gas supply scheduling strategies.

## 4. Experiment

### 4.1. Dataset Description and Preprocessing

The raw data used in this study primarily originates from a branch of a natural gas station in Hanzhong, China, spanning historical daily load data from 2016 to 2022. Hanzhong City is situated in southern China. Characterized by a mild and humid climate, it occupies a unique geographical position that links the Guanzhong Plain urban cluster and the Chengdu–Chongqing urban cluster, playing a crucial role in the regional economy. To accurately predict NGL, this study also collected meteorological data from the Hanzhong station of the National Centers for Environmental Information in the United States, located at coordinates (107.02, 33.04). The specific factors include average temperature (TEMP), average dew point (DEWP), average visibility (VISIB), average wind speed (WDSP), maximum sustained wind speed (MXSPD), maximum temperature (MAXT), minimum temperature (MINT), and precipitation (PRCP). Additionally, this study integrates date type (DT) and seasonal type (ST) factors.

**Figure 3:**
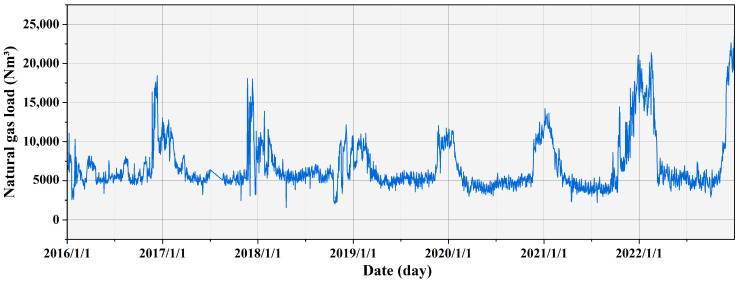
Natural gas daily load data.

Due to objective reasons causing data gaps, this study employed linear spline interpolation to fill in missing values. Distinct linear interpolation strategies were applied to intervals with different missing values to achieve smooth data completion. The processed historical daily load data, as shown in Figure 3, yielded a total of 2557 samples. The descriptive statistical analysis of all data is presented in Table 2, encompassing maximum values, minimum values, mean values, standard deviations, medians, variances, kurtosis, skewness, and coefficients of variation. The standard deviation and variance of NGL data are large, indicating a high degree of dispersion and variability among the data points. A kurtosis above 3 indicates that the data are distributed more sharply. With a skewness of 2.066, the data exhibit a rightward deviation from its mean. The use of the augmented Dickey–Fuller test further confirms the stationarity of NGL data, providing a reliable foundation for subsequent time series analysis, as depicted in Table 3. The *p*-value of the series is 0.096, which is greater than 0.05, indicating that the series has a unit root and is non-stationary.

### 4.2. Analysis of Complexity Features

This paper analyzes the complex features of historical data, including FD, HE, SE, and MLE. These features capture the self-similarity, long-term memory, randomness, and chaos features in NGL.

**Figure 4:**
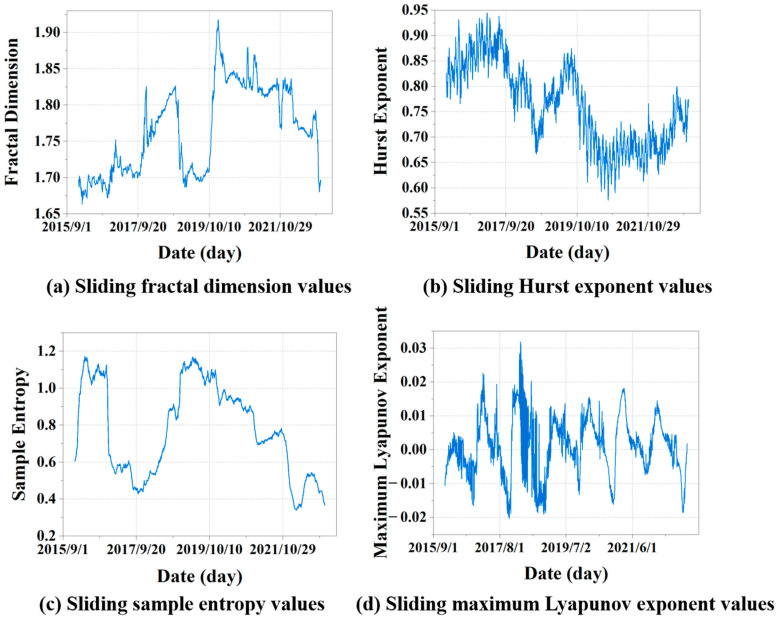
Sliding fractal dimension, Hurst exponent, sample entropy, and maximum Lyapunov exponent of NGL.

Appropriate sliding windows facilitate efficient computation and extraction of internal information patterns within the data. The computation of complex data features typically requires long time series data, taking into account the seasonality and periodicity of the data. The window size should exceed the average autocorrelation time of the data to fully account for its long-term correlations. Natural gas historical load data exhibit an annual cyclic pattern with similarities between consecutive years. Hence, this study sets the window size to 365 data points. The specific results of complexity feature calculations are illustrated in Figure 4.

Figure 4a shows that the sliding window calculation of the FD yields a maximum value of 1.9179 and a minimum value of 1.6633, indicating a relatively high level of FD. The results indicate that the sliding time series exhibits strong self-similarity and complex dynamic behavior, characterized by multi-scale features arising from fluctuations at various time scales. Complex models are required for this type of data to capture data characteristics and predict future changes.

Figure 4b presents the sliding-window calculation results of the HE, with a maximum value of 0.9450 and a minimum of 0.5760. An HE value approaching 0.5 is typically interpreted as indicative of a random sequence. Values greater than 0.5 indicate positive long-term correlations in the sequence, reflecting varying degrees of long-term memory in the time series. In this scenario, the data exhibits some trendiness, suggesting that current changes are likely to persist into the future for some time.

As shown in Figure 4c, the maximum sliding value of SE is 1.1703, while the minimum is 0.3383. A high SE value indicates that the data has a high degree of irregularity and randomness. Low SE values indicate data with high regularity and relative simplicity. In the natural gas daily load forecasting data analyzed in this study, a value around 0.3383 is relatively low. Values approaching or exceeding 1.0 are considered high SE. Different time scales reveal varying levels of complexity and patterns of change, indicating stochastic variability in the data’s patterns.

Figure 4d presents the results of the sliding MLE calculation. The maximum value observed is 0.0318, while the minimum is −0.0204. A positive MLE indicates that the data may exhibit chaotic properties, while a negative value suggests that the sequence tends toward stability. For NGL, positive values indicate that the system exhibits uncertainty and variability during certain periods. Although the rate of change is relatively low, it still exerts a noticeable influence on the long-term dynamics of the series. In this context, NGL may be influenced by factors such as seasonality, supply and demand dynamics, or operational activities at facilities. Negative values indicate that the NGL exhibits stability and convergence over a certain period. At this point, the system can cope with external influences and can regulate.

Additionally, this section also calculates the overall complexity characteristics of historical NGL. The result for FD is 1.753, indicating that the load exhibits similar statistical distribution properties over time scales. The HE value of 0.954 indicates that the load tends to maintain similar trends over a longer time range, suggesting a certain level of correlation between successive states. The SE value of 0.5965 indicates a significant degree of randomness and uncertainty within the time series. The MLE is 0.0118, a tiny positive value indicating that the system may have some dynamic changes and chaos. These results provide significant statistical references for the characteristics of daily NGL.

### 4.3. Ranking of Feature Importance

Feature importance ranking holds significant importance in predictive tasks. By analyzing and determining the extent to which each feature contributes to the predicted outcome, one can gain a deeper understanding of the critical driving factors within the data. It is important to note that for data with significant volatility, the influence of nonlinear factors needs to be paid attention to. Tree-based ensemble embedding methods are effective in conducting nonlinear correlation analysis and have demonstrated notable success in feature selection tasks [40]. These methods are capable of outputting feature importance rankings upon completion of model training [17]. Previous studies on NGL forecasting have not considered data features such as FD, HE, SE, and MLE. The results of the complexity analysis are fused into the traditional feature information in this section to constitute a new feature dataset, allowing the forecasting model to have sufficient auxiliary information in understanding the load data. The total feature set can be found in Supplementary Materials Table S1.

**Figure 5:**
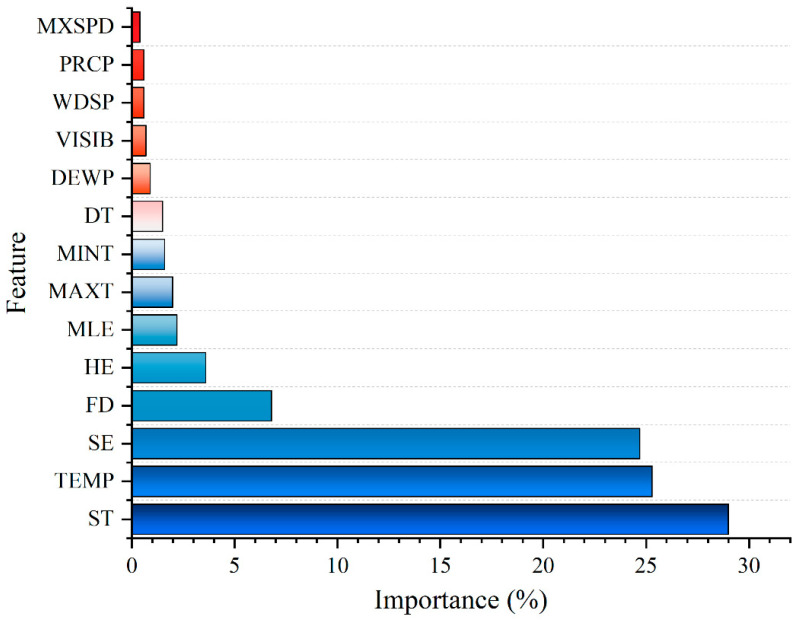
Ranking of feature importance.

This section focuses on conducting importance ranking of TEMP, DEWP, VISIB, WDSP, MXSPD, MAXT, MINT, PRCP, DT, ST, FD, HE, SE, and MLE using XGBoost. The results of feature importance are presented in Figure 5. The importance of complexity features is observed to be second only to ST and TEMP. Based on the calculated feature importance results, this section proposes to select features with importance metrics exceeding 1.5% for further in-depth investigation as key features (marked in blue). Specifically, these include ST, TEMP, FD, HE, SE, MLE, MAXT, and MINT. Using XGBoost for feature importance ranking across a series of external influencing factors and data complexity characteristics ensures the effectiveness of input features, mitigates redundancy, and enhances predictive performance.

### 4.4. Data Decomposition

**Figure 6:**
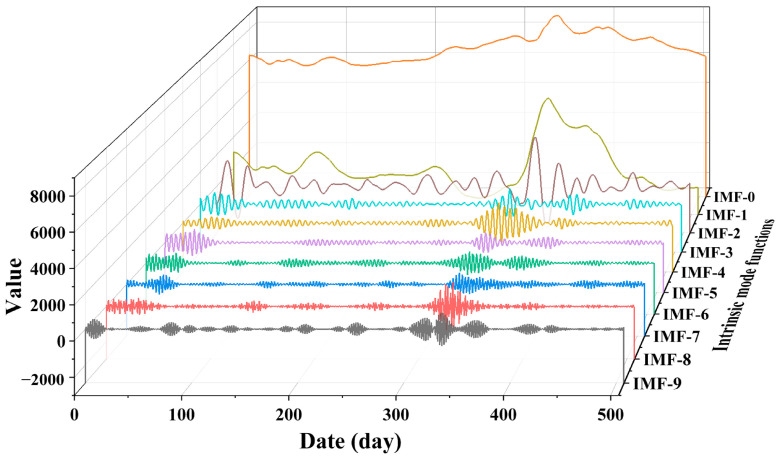
Intrinsic mode functions (IMF0~IMF9).

This section utilizes VMD to decompose the complex load data into several intrinsic mode functions (IMF0 to IMF9). The VMD method provides a clearer and more interpretable modal decomposition, as shown in the detailed results in Figure 6. IMF0 to IMF9 represent distinct frequency components of NGL fluctuation patterns. The decomposition results reveal distinct fluctuation characteristics of each modal component in the time series, arranged from high to low frequencies. This reflects the complexity and diversity of fluctuations in NGL data. Each modal component exhibits pronounced periodic or trend-based variations, indicating the temporal dynamics of NGL data across different time scales.

### 4.5. Evaluation Indicators

This study analyzes the performance of load forecasting using four widely adopted evaluation metrics [4]: mean absolute percentage error (MAPE), root mean squared error (RMSE), mean absolute error (MAE), and coefficient of determination (R^2^). The specific definitions are presented in Table 4.

### 4.6. Experimental Results and Discussions

#### 4.6.1. Application and Comparison of Complexity Features

This study incorporates complexity analysis into the scope of feature considerations, with a detailed feature set outlined in Supplementary Materials Table S1. These features encompass not only traditional meteorological factors but also complex features such as self-similarity, long-term memory, randomness, and chaos features.

As indicated in Section 4.2, key features identified through XGBoost for feature importance ranking include ST, TEMP, FD, HE, SE, MLE, MAXT, and MINT. This analysis considers four complexity features, each offering valuable insights into the underlying dynamics of the time series. Furthermore, they offer theoretical support for forecasting and managing future load demands, bearing significant relevance in predicting future trends.

To validate the feature screening effect of the XGBoost model, this study designed multiple distinct feature datasets as inputs for detailed comparative analysis. As shown in Table 5, these include the unfiltered feature set, the set comprising only complexity analysis features, the set containing only meteorological factors, and the crucial feature set identified through XGBoost selection.

**Figure 7:**
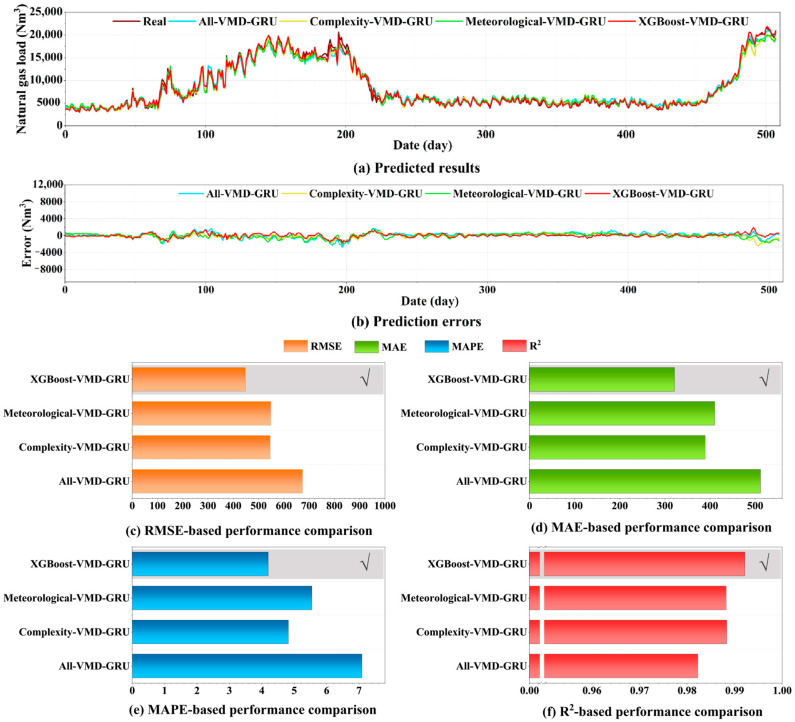
Comparison of the forecasting performance of hybrid models combining different feature sets with the VMD-GRU model. The checkmark (√) in (**c**–**f**) indicates the model with the best prediction performance.

The combined predictive performance of hybrid models integrating various feature sets with the VMD-GRU model is illustrated in Figure 7a. The figure demonstrates that the predictive trends of the All-VMD-GRU, Complexity-VMD-GRU, Meteorological-VMD-GRU, and XGBoost-VMD-GRU hybrid models closely resemble the actual values, indicating that VMD-GRU effectively learns from historical load data. The XGBoost-VMD-GRU model, which considers complexity features, exhibits the highest degree of overlap with the actual values, indicating its superior predictive capability. The error results shown in Figure 7b indicate that the red line (XGBoost-VMD-GRU) fluctuates slightly around zero, suggesting relatively low prediction errors.

Furthermore, prediction performance metrics were calculated to assess the model’s accuracy. As illustrated in Figure 7c–f, the proposed model outperforms the comparative models in terms of RMSE, MAE, MAPE, and R^2^. Evaluating predictive performance metrics can reflect the prediction accuracy of the models. As shown at the bottom of Figure 7, the proposed models demonstrate the highest predictive accuracy. Additionally, Table 6 illustrates significant differences in predictive performance among different feature sets. Overall, the XGBoost-VMD-GRU model that incorporates complexity features exhibited optimal predictive performance with an RMSE of 447.914292 Nm^3^, MAE of 321.934698 Nm^3^, MAPE of 4.200876%, and R^2^ of 0.992201. In contrast, the All-VMD-GRU model shows the lowest level of predictive accuracy. Complexity-VMD-GRU and Meteorological-VMD-GRU produce comparable results; however, both remain less accurate than the XGBoost-VMD-GRU model, which demonstrates notably higher performance. Experimental results demonstrate that integrating complexity features into the feature set and efficiently selecting the relevant data through XGBoost significantly improves prediction accuracy during the forecasting process.

#### 4.6.2. Comparison of Different Models

**Figure 8:**
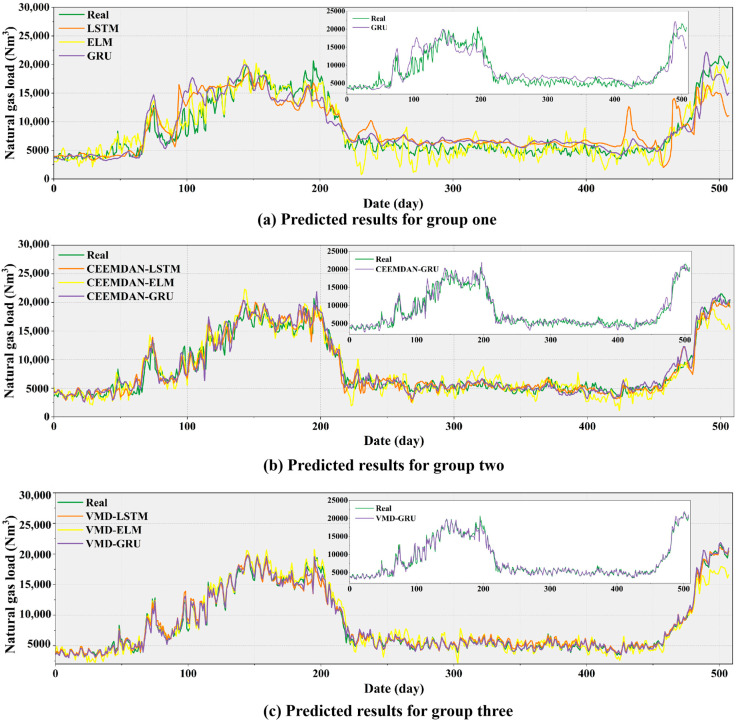
Results of predictions for multiple comparative experiments.

To further prove the validity of the proposed hybrid model, multiple ablation experiments are conducted in this section for a comprehensive comparison. Specifically, this section employs extreme learning machine (ELM), LSTM, and GRU as foundational prediction models, with CEEMDAN and VMD utilized as signal decomposition methods. For the prediction of daily NGL, three sets of experiments were designed. Group one includes ELM, LSTM, and GRU. Group two consists of CEEMDAN-ELM, CEEMDAN-LSTM, and CEEMDAN-GRU. Group three comprises VMD-ELM, VMD-LSTM, and VMD-GRU. Note that this section uses feature data filtered by XGBoost and historical loadings for validation. The forecasting results in Figure 8 illustrate the outcomes of the three experimental groups. The three plots in Figure 8a–c clearly illustrate the predictive trends. In Group Three, where VMD is used for decomposition and GRU for forecasting, the predicted curve shows strong agreement with the actual values.

The detailed prediction evaluation results are presented in Table 7, illustrating that the decomposed prediction models outperform single models across multiple metrics. The adoption of decomposition methods significantly enhances prediction accuracy, with the decomposition effectiveness of VMD notably superior to that of CEEMDAN. VMD effectively extracts periodic and trend components from the data, providing more accurate input information for the model. For instance, the GRU model enhanced by VMD demonstrates a reduction of 17.8570% in MAPE compared to the standalone GRU model. Similarly, RMSE and MAE metrics also exhibit reductions. Notably, although the R^2^ value of the single model did not exceed 90%, all decomposition-based prediction models outperformed this benchmark. In particular, the VMD-GRU model achieved a remarkably high R^2^, exceeding 99%. Thus, the decomposed prediction model, particularly VMD-GRU, demonstrates significant competitiveness and superiority across multiple evaluation metrics. The detailed experimental results can be found in Supplementary Materials Table S2. Additionally, Section S2 of the Supplementary Materials presents a comparative discussion between the proposed model and three recent time series forecasting models.

#### 4.6.3. Comparison of Different Decomposition Approaches

**Figure 9:**
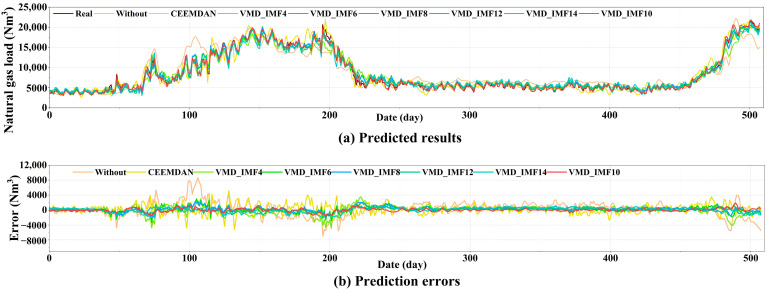
Prediction and error results for different decomposition methods combined with XGBoost and GRU.

The intrinsic mode number of VMD in this study is determined with reference to the adaptive decomposition results of CEEMDAN, and through multiple experiments, an optimal number of 10 layers has been identified. To validate the prediction effectiveness of the VMD_IMF10 method, this section contrasts various decomposition methods and IMF quantities. The specific decomposition methods include no decomposition, CEEMDAN, VMD_IMF4, VMD_IMF6, VMD_IMF8, VMD_IMF10, VMD_IMF12, and VMD_IMF14. Based on the key features selected by XGBoost discussed in Section 4.6.1, experiments were conducted to compare prediction and error results obtained from different combinations of decomposition methods with GRU, as illustrated in Figure 9. Figure 9a shows that the prediction results obtained using VMD_IMF10 are closest to the actual values. Figure 9b illustrates that the error lines are distributed around zero, indicating that decomposition with VMD_IMF10 achieves the best performance.

**Figure 10:**
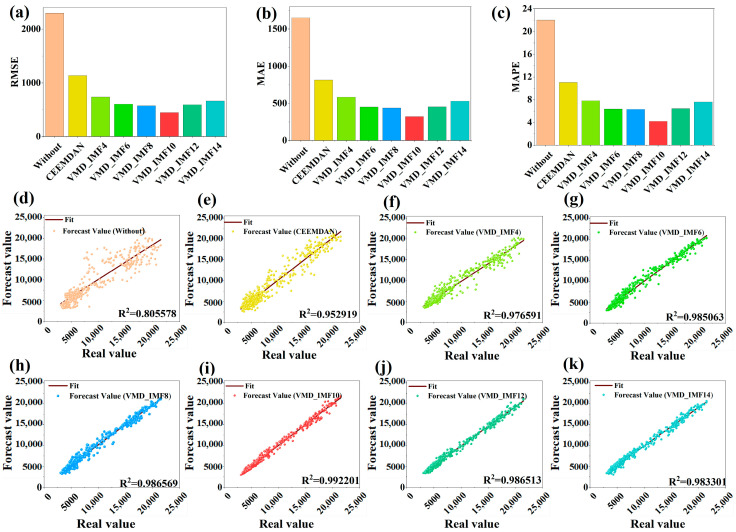
Evaluation results of the prediction performance of different decomposition methods. Subfigures (**a**–**c**) compare the RMSE, MAE, and MAPE metrics under different decomposition methods combined with XGBoost and GRU. Subfigures (**d**–**k**) present scatter plots of predicted versus actual values.

Figure 10 presents the prediction performance results of this experiment. Subplots (a), (b), and (c) compare the RMSE, MAE, and MAPE metrics under different decomposition methods combined with XGBoost and GRU. The results indicate that VMD_IMF10 achieves the lowest values across all three metrics. In addition, Figure 10d–k present scatter plots of predicted versus actual values. Among them, the results from the VMD_IMF10 decomposition show the best fit, as indicated by the R^2^ values closest to 1. Therefore, decomposing the sequence into ten IMFs using the VMD method is identified as the optimal approach. The corresponding predictive evaluation results are shown in Table 8. The results indicate that the optimal VMD_IMF10 achieves an RMSE of 447.914291, an MAE of 321.934698, a MAPE of 4.2008763%, and an R^2^ value of 0.992201.

#### 4.6.4. Discussion of Different Prediction Steps

**Figure 11:**
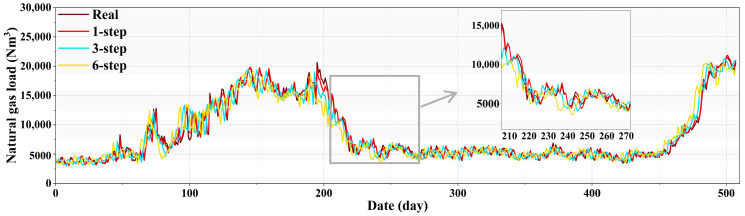
Diagram of multi-step prediction results of XGBoost-VMD-GRU considering complexity features.

The selection of prediction time steps directly influences the forecast accuracy and practical utility of the model in predicting future load variations. To evaluate the multi-step forecasting performance of the proposed model, this section configures the prediction input for 12 steps with output steps of 6. The code execution records predictions at intervals of one, three, and six steps, as illustrated in Figure 11. The experiments indicate that the one-step forecasting results closely match the actual data. According to the data in Table 9, the R^2^ for one-step prediction is 0.992201, for three-step prediction is 0.986031, and for six-step prediction is 0.967999. Overall, the results are above 0.95, indicating that the proposed target model predicts well.

In this section, in addition to conducting multi-step prediction experiments on the proposed model, we also perform multi-step natural gas daily load forecasting experiments using different feature datasets proposed in this paper (All, Complexity analysis only, Meteorological factor only, and XGBoost screened) and multiple comparative models (ELM, LSTM, GRU, CEEMDAN-ELM, CEEMDAN-LSTM, CEEMDAN-GRU, VMD-ELM, VMD-LSTM, and VMD-GRU). Supplementary Materials Table S3 presents the performance evaluation results of RMSE, MAE, MAPE, and R^2^ for all experiments at one-step, three-step, and six-step predictions. The experimental results indicate significant variations in performance across different models, datasets, and prediction horizons.

**Figure 12:**
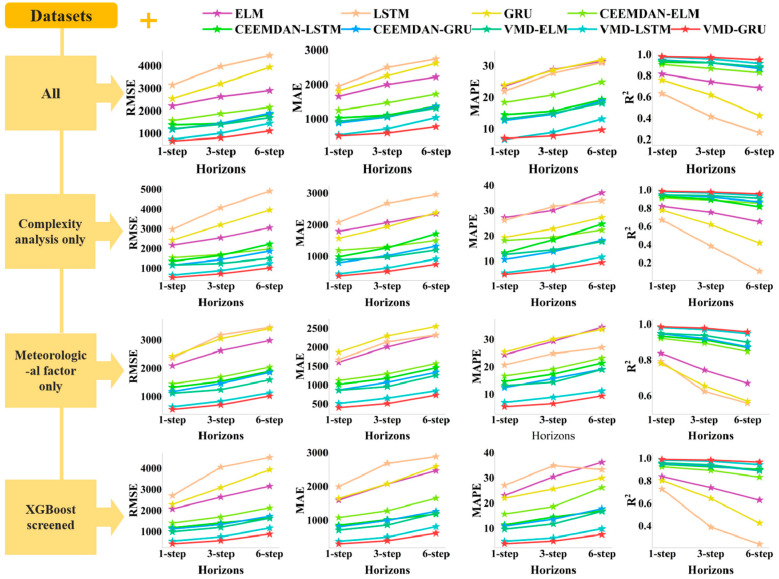
The RMSE, MAE, MAPE, and R^2^ metrics for one-step, three-step, and six-step forecasts are presented for various comparative models (including ELM, LSTM, GRU, CEEMDAN-ELM, CEEMDAN-LSTM, CEEMDAN-GRU, VMD-ELM, VMD-LSTM, and VMD-GRU) across different feature datasets.

To enhance visual clarity, the predictive performance of all experiments is summarized in Figure 12. This figure consists of 16 subplots arranged in a 4 × 4 grid. Each column represents one of the four evaluation metrics: RMSE, MAE, MAPE, and R^2^. Each row corresponds to a different feature selection method used to construct the input dataset for the forecasting tasks. Within each subplot, the performance of the nine models considered in this study is presented under one-step, three-step, and six-step prediction scenarios. Generally, multi-step forecasting requires consideration of increased uncertainty in future periods compared to one-step forecasting, thereby being more susceptible to cumulative error and presenting greater forecasting challenges. Therefore, in terms of overall predictive performance, one-step > three-step > six-step. In comparing the predictive performance across multiple models, decomposition prediction models outperform single prediction models, with the VMD-GRU model showing particularly superior performance. For the four datasets, the complexity features and meteorological features screened by XGBoost combined with the load data had the best prediction performance.

In practical applications, one-step forecasting provides immediate and precise load prediction results, which are crucial for natural gas scheduling and market transactions. However, for decisions such as long-term resource planning and gas supply arrangements, three-step and six-step forecasts become more critical. These forecasts capture trends over longer time spans, aiding in the formulation of more sustainable strategies to meet evolving natural gas demands and market conditions.

#### 4.6.5. Robustness Analysis

To explore the robustness of XGBoost-VMD-GRU in real-world applications, this section conducts robustness analysis by introducing various noise distributions to the original test set. By comparing the performance of the proposed models under various noise conditions, one can assess their robustness against anomalous data and thereby enhance the reliability of these models in practical applications. The high robustness of the model implies its ability to maintain stable performance in the presence of disturbances, thereby enhancing the reliability of its applications [41].

In this experiment, Gaussian distribution $N(0,\sigma^{2})$, Poisson distribution P(λ), uniform distribution *U*(a,b), and exponential distribution EXP(λ) were introduced as noise into the data to simulate various disturbances that may occur in real-world scenarios. Meanwhile, different parameters were tuned for each type of noise to evaluate the performance of the model under different levels of interference, and the specific results are shown in Table 10, Table 11, Table 12 and Table 13. Experimental results demonstrate that, despite the introduction of noise into the data, the predictive performance of the XGBoost-VMD-GRU model that incorporates complexity features experiences only a slight decline. The overall R^2^ remains above 0.986, indicating its exceptional predictive capability. The most significant performance degradation was observed under Gaussian noise with a variance of 2. Even in this case, the R^2^ value decreased by only 0.005996, indicating strong robustness of the proposed model.

#### 4.6.6. Statistical Tests

In this section, the Diebold–Mariano test was employed to further validate the scientific rigor and reliability of the proposed model. The DM test is fundamentally a *t*-test [42]. It is used to determine whether there exists a statistically significant difference in prediction errors between two forecasting models. In contrast to traditional error evaluation metrics, it provides a rigorous statistical comparison based on empirical errors, offering a more comprehensive assessment of the relative strengths and weaknesses of different models in prediction. The optimal dataset filtered by XGBoost was used for the experiments in this section (see Section 4.6.1). The predictive effectiveness of VMD-GRU, accounting for data complexity, was compared with eight models (ELM, LSTM, GRU, CEEMDAN-ELM, CEEMDAN-LSTM, CEEMDAN-GRU, VMD-ELM, and VMD-LSTM) using the DM test. Detailed results are presented in Table 14. In all cases, the null hypothesis assumed that the two models have identical predictive performance. However, all *p*-values were less than 0.01, indicating rejection of the null hypothesis at the 1% significance level. This suggests significant differences in predictive performance between VMD-GRU and the eight benchmark models. Furthermore, all DM values were negative, indicating that VMD-GRU exhibited significantly lower prediction errors compared to the other models. The extremely small *p*-values further confirm that the superiority of VMD-GRU observed in the experiments is not incidental but attributable to significant differences in its design and performance. Therefore, it can be concluded that VMD-GRU outperforms the compared models significantly in terms of performance.

## 5. Conclusions and Future Directions

Historical loadings of natural gas are characterized by complexity. Achieving more accurate forecasts requires a deep understanding of the complex characteristics of historical sequences and their impact on predictability. However, existing research rarely incorporates the complex features of historical load data into natural gas forecasting frameworks. Therefore, this paper proposes a load-forecasting model based on data decomposition and ensemble deep learning that combines data complexity features.

This study employs FD, HE, SE, and MLE to analyze the self-similarity, long-term memory, randomness, and chaotic features of historical NGL data, addressing the data complexity features and exploring the complexities of time series. Next, employing XGBoost, we conducted an importance ranking of four complexity features and ten collected meteorological features, identifying eight key features. Subsequently, VMD decomposed the load data into ten intrinsic mode functions (IMFs). These modes were integrated with selected key features as input vectors for the GRU network to perform multi-step forecasting. Furthermore, this study discusses the predictive performance of various feature datasets and different combinatorial models. The results further validate the efficiency of the hybrid XGBoost-VMD-GRU model considering data complexity features in multi-step NGL forecasting. To better meet practical application needs, this study conducts multi-step forecasting experiments for daily NGL using various feature datasets and comparative models. This approach aids in formulating sustainable strategies for natural gas resource planning and supply scheduling. Finally, this study performs robustness analysis and the DM test on the proposed methods, comprehensively demonstrating their superiority across various metrics.

The hybrid predictive model that accounts for data complexity features can be applied in energy load forecasting. This study validates the reliability of the method using data from Hanzhong, Shaanxi. The research findings will aid in the optimization of the current energy forecasting framework and provide valuable insights for energy demand management and market operations. Additionally, this research can serve as a theoretical reference for other fields, particularly those involving the analysis and forecasting of complex time series data, such as financial markets and environmental science.

Despite the strong performance of the proposed model, several limitations remain to be addressed. (1) Potential overfitting risk: although the model achieves high accuracy on the current dataset, it may overfit when trained on limited data. Future work will explore regularization techniques and cross-validation strategies to reduce this risk. (2) Limited regional transferability: the model has not been extensively tested in regions with different natural gas consumption patterns. Its generalizability across regions needs further validation. Subsequent research will conduct transferability assessments incorporating multi-source data to expand the model’s application scope. (3) Sensitivity to noise: this study conducted an initial robustness analysis by introducing Gaussian, Poisson, uniform, and exponential noise, demonstrating the model’s stability under certain perturbations. However, the model may still exhibit performance fluctuations when dealing with unstructured or extremely anomalous data. Subsequent work will consider integrating robust optimization methods or anomaly detection mechanisms to improve adaptability under complex data environments. These limitations suggest clear directions for future research and can support more reliable and scalable applications of the model in real-world energy systems.

## Figures and Tables

**Table 1:** Summary of methods for data complexity analysis.

Methods	Abbreviations	Description	Typical Value Range	Practical Implications
Fractal Dimension	FD	It quantifies the self-similarity of a sequence to facilitate a deeper comprehension of the dynamic characteristics and behavioral patterns inherent in the data.	(1, 2)	A larger value indicates a rougher and more complex sequence.
Hurst Exponent	HE	It quantifies long-term memory and persistence characteristics within the sequence.	(0, 1)	HE < 0.5 indicates an anti-persistent sequence; HE > 0.5 signifies a persistent sequence; and HE = 0.5 denotes a random walk sequence.
Sample Entropy	SE	It quantifies the degrees of randomness and uncertainty inherent in the data.	[0, +∞)	A larger value indicates a higher degree of disorder and greater unpredictability within the sequence.
Maximum Lyapunov Exponent	MLE	It captures the system’s sensitivity to initial condition perturbations, characterizing chaotic behavior.	(−∞, +∞)	A positive value of the data suggests the potential presence of chaotic characteristics, whereas a negative value indicates that the sequence tends toward stability.

**Table 2:** Descriptive statistical analysis of the dataset.

NO.	Data	Sample Size	Maximum	Minimum	Mean	Standard Deviation	Median	Variance	Kurtosis	Skewness
1	TEMP	2557	93.8	27.1	60.956	15.545	61.1	241.647	−1.158	−0.031
2	DEWP	2557	80.4	−2.1	51.821	15.242	53.3	232.328	−0.883	−0.272
3	VISIB	2557	18.6	0.9	11.106	4.956	11.3	24.564	−1.129	−0.171
4	WDSP	2557	8.7	0.2	2.193	0.781	2.1	0.61	7.863	1.877
5	MXSPD	2557	15.5	1.4	3.705	1.483	3.9	2.199	3.762	1.22
6	MAXT	2557	104.7	28.4	69.135	16.821	69.3	282.952	−1.069	−0.057
7	MINT	2557	83.8	17.2	53.68	15.088	54.1	227.639	−1.168	−0.084
8	PRCP	2557	4.11	0	0.11	0.318	0	0.101	37.782	5.312
9	DT	2557	2	0	0.391	0.627	0	0.393	0.677	1.36
10	ST	2557	0.8	0.2	0.498	0.223	0.4	0.05	−1.358	0.011
11	DNGL	2557	23,510.52	1548	6800.324	3349.295	5614	11,217,775.41	4.586	2.066

**Table 3:** Stationarity test.

	t	*p*	Critical Value		
			1%	5%	10%
DNGL	−2.587	0.096	−3.433	−2.863	−2.567

**Table 4:** Evaluation metrics.

	Metrics	Formula	Rules
1	MAPE	$MAPE=\frac{1}{N}\sum_{t=1}^{N}\left|\frac{A_{t}-F_{t}}{A_{t}}\right|\times 100\%$	The smaller, the more accurate.
2	RMSE	$RMSE=\sqrt{\frac{1}{N}\sum_{t=1}^{N}{\left(A_{t}-F_{t}\right)}^{2}}$	The smaller, the more accurate.
3	MAE	$MAE=\frac{1}{N}\sum_{t=1}^{N}\left|A_{t}-F_{t}\right|$	The smaller, the more accurate.
4	$R^{2}$	$R^{2}=1-\frac{\sum_{t=1}^{N}{\left(F_{t}-A_{t}\right)}^{2}}{\sum_{t=1}^{N}{\left(\bar{A_{t}}-A_{t}\right)}^{2}}$	$R^{2}$$\;\mathrm{is}\;\mathrm{between}\;0\;\mathrm{and}\;1;\;\mathrm{the}\;\mathrm{closer}\;R^{2}$ is to 1, the more accurate it is.

Notes: *N* is the length of the data; $A_{t}$ is the actual value; $F_{t}$ is the predicted value; $\bar{A_{t}}$ is the average of the actual value; and $\bar{F_{t}}$ is the average of the predicted value.

**Table 5:** Different ways of filtering features and details of feature sets.

Feature Set	Description	Detail
All	A complete set of unfiltered features.	TEMP, DEWP, VISIB, WDSP, MXSPD, MAX, MIN, PRCP, DT, ST, HE, SE, FD, MLE
Complexity analysis only	Feature sets that contain only complexity analysis.	HE, SE, FD, MLE
Meteorological factor only	Feature sets containing only weather, date type, and season type.	TEMP, DEWP, VISIB, WDSP, MXSPD, MAX, MIN, PRCP, DT, ST
XGBoost screened	Key feature sets selected by XGBoost.	TEMP, MAX, MIN, ST, HE, SE, FD, MLE

**Table 6:** Results of forecast evaluation of different feature sets combined with VMD-GRU.

Model	RMSE	MAE	MAPE	$R^{2}$
All-VMD-GRU	674.488371	512.518293	7.095566	0.982316
Complexity-VMD-GRU	546.501682	389.877773	4.824470	0.988391
Meteorological-VMD-GRU	549.066995	411.110791	5.549112	0.988281
XGBoost-VMD-GRU	447.914292	321.934698	4.200876	0.992201

**Table 7:** Results of predictive evaluation of multiple comparative experiments.

Feature Set	Metrics	Single Forecasting Model			Decomposition Forecasting Model					
		ELM	LSTM	GRU	CEEMDAN-ELM	CEEMDAN-LSTM	CEEMDAN-GRU	VMD-ELM	VMD-LSTM	VMD-GRU
XGBoost screened	RMSE	2086.331549	2716.918223	2300.972869	1418.953999	1191.727077	1134.998723	1005.942378	577.876172	447.914292
	MAE	1602.132318	2002.254127	1651.114807	1088.172136	861.348212	814.350885	717.038583	398.331519	321.934698
	MAPE	23.151721	27.155891	22.057922	15.811080	11.542723	11.044376	9.808534	5.202687	4.200876
	$R^{2}$	0.840159	0.728934	0.805578	0.926330	0.948037	0.952919	0.960666	0.987019	0.992201

**Table 8:** Results of prediction evaluation for different decomposition methods.

Metrics	Decomposition Method							
	No Decomposition	CEEMDAN	VMD_IMF4	VMD_IMF6	VMD_IMF8	VMD_IMF10	VMD_IMF12	VMD_IMF14
RMSE	2300.972869	1134.998723	738.2005945	606.115283	576.013733	447.914291	594.157103	663.385375
MAE	1651.114807	814.350885	584.798344	453.206701	438.779238	321.934698	454.483538	530.699079
MAPE	22.057922	11.044376	7.834173	6.373846	6.343959	4.2008763	6.444841	7.630316
$R^{2}$	0.805578	0.952919	0.976591	0.985063	0.986569	0.992201	0.986513	0.983301

**Table 9:** The performance results of XGBoost-VMD-GRU with multi-step prediction considering complexity features.

	Step Size	RMSE	MAE	MAPE	$R^{2}$
XGBoost-VMD-GRU	One step	447.914292	321.934698	4.200876	0.992201
	Three steps	599.747286	414.108263	5.204439	0.986031
	Six steps	905.755273	630.943584	7.787919	0.967999

**Table 10:** Results of prediction performance after adding Gaussian distribution noise.

$N(0,\sigma^{2})$	RMSE	MAE	MAPE	$R^{2}$
Normal	447.914292	321.934698	4.200876	0.992201
σ = 0.2	492.635181	348.182688	4.508906	0.990566
σ = 0.4	516.884271	372.973968	4.909615	0.989615
σ = 0.6	550.019130	413.243982	5.537968	0.988241
σ = 0.8	561.713045	415.665926	5.936531	0.987735
σ = 1	560.878278	414.557184	5.533741	0.987772
σ = 2	595.731438	430.793072	5.282246	0.986205

**Table 11:** Results of prediction performance after adding Poisson distribution noise.

P(λ)	RMSE	MAE	MAPE	$R^{2}$
Normal	447.914292	321.934698	4.200876	0.992201
λ = 1	451.156046	327.541571	4.344449	0.992088
λ = 2	478.850837	364.327628	5.185826	0.991087
λ = 4	482.163025	351.250970	4.693562	0.990964
λ = 8	506.049992	367.757744	5.020558	0.990046
λ = 16	532.947304	395.211437	5.489871	0.988960

**Table 12:** Results of prediction performance after adding uniform distribution noise.

U (a, b)	RMSE	MAE	MAPE	$R^{2}$
Normal	447.914292	321.934698	4.200876	0.992201
$\left(a,\;b\right)=(-0.5, 0.5)$	509.954158	374.739492	5.218619	0.989892
$\left(a,\;b\right)=(-1, 1)$	550.655201	392.648943	4.892983	0.988214
$\left(a,\;b\right)=(-2, 2)$	531.684776	400.900071	5.766418	0.989012
$\left(a,\;b\right)=(-5, 5)$	530.323694	401.832223	5.678757	0.989068
$\left(a,\;b\right)=(-10, 10)$	543.076969	418.183454	6.050953	0.988537

**Table 13:** Results of prediction performance after adding exponential distribution noise.

EXP(λ)	RMSE	MAE	MAPE	$R^{2}$
Normal	447.914292	321.934698	4.200876	0.992201
λ = 1	492.356910	361.274683	4.866360	0.990577
λ = 2	484.870815	349.839395	4.670203	0.990862
λ = 4	489.147496	374.501924	5.356099	0.990699
λ = 8	495.410941	370.421822	5.159385	0.990460
λ = 16	501.701613	361.887091	4.730034	0.990216

**Table 14:** Diebold–Mariano test results between VMD-GRU and other models, respectively.

Model (Consider the Complexity of the Data)	DM Value	*p* Value
ELM	−12.970941 ***	1.992764 × 10^−33^
LSTM	−12.153916 ***	5.241625 × 10^−30^
GRU	−10.360662 ***	6.044957 × 10^−23^
CEEMDAN-ELM	−11.057308 ***	1.308850 × 10^−25^
CEEMDAN-LSTM	−9.867247 ***	3.998776 × 10^−21^
CEEMDAN-GRU	−9.127753 ***	1.655615 × 10^−18^
VMD-ELM	−8.464992 ***	2.772544 × 10^−16^
VMD-LSTM	−4.632283 ***	4.603091 × 10^−6^

*** denotes significance levels at 1%.

## Data Availability

The data presented in this study are available on request from the corresponding author.

## Supplementary Materials

The following supporting information can be downloaded at: https://www.mdpi.com/article/10.3390/e27070671/s1. References [43,44,45] are cited in the Supplementary Materials.

## Nomenclature

Abbreviations	
CEEMDAN	Complete Ensemble Empirical Mode Decomposition with Adaptive Noise
DT	Date Type
DEWP	Average Dew Point
ELM	Extreme Learning Machine
FD	Fractal Dimension
GA	Genetic Algorithm
GRU	Gate Recurrent Unit
HE	Hurst Exponent
IMFs	Intrinsic Mode Functions
LSTM	Long Short-Term Memory
MAE	Mean Absolute Error
MAPE	Mean Absolute Percentage Error
MAXT	Maximum Temperature
MINT	Minimum Temperature
ML	Machine Learning
MLE	Maximum Lyapunov Exponent
MXSPD	Maximum Sustained Wind Speed
NGL	Natural Gas Load
PRCP	Precipitation
ReLU	Rectified Linear Unit
RMSE	Root Mean Squared Error
R^2^	Coefficient of Determination
R/S analysis	Rescaled Range analysis
SE	Sample Entropy
SELU	Scaled Exponential Linear Unit
ST	Seasonal Type
SVR	Support Vector Regression
TEMP	Average Temperature
VISIB	Average Visibility
VMD	Variational Mode Decomposition
WDSP	Average Wind Speed
XGBoost	eXtreme Gradient Boosting
Variables	
$X_{k}^{m}$	A subsequence set partitioned from the starting point *m* with a delay interval *k*.
$L_{m}\left(k\right)$	Curve length of the subsequence starting at an initial time point *m* with a time interval *k*.
$L\left(k\right)$	The mean curve length at identical time intervals *k*.
$\bar{X_{a}}$	The mean value of subsequence *a*.
$Y_{ab}$	For subsequence *a*, the cumulative deviations are calculated progressively for the initial *b* data points.
$R_{a}$	Range of subsequence *a*.
$S_{a}$	Standard deviation of subsequence *a*.
$u_{k}$	*k*-th intrinsic mode function.
$\omega_{k}$	Central frequency of the *k*-th mode.
$r_{t}$	Reset gate of the GRU.
$z_{t}$	Update gate of the GRU.
${\tilde{h}}_{t}$	Candidate hidden state of the GRU.
$h_{t}$	Final hidden state of the GRU.
