# Supplementary material for: Multi-Step Natural Gas Load Forecasting Incorporating Data Complexity Analysis with Finite Features
Source: Entropy (Basel). 2025 Jun 23;27(7):671. doi: 10.3390/e27070671 (PMC12293877; doi:10.3390/e27070671)
Supplement: Supplementary file 1 [file entropy-27-00671-s001.zip › entropy-3659008-supplementary.pdf]

Multi-Step Natural Gas Load Forecasting Incorporating Data Complexity Analysis with Finite Features

Supplementary Materials

S1. Supplementary tables

Table S1 The total set of features.

	Feature	Value	Units
Meteorological features	TEMP	[27.1, 93.8]	°F
	DEWP	[-2.1, 80.4]	°F
	VISIB	[0.9, 18.6]	mile
	WDSP	[0.2, 8.7]	knot
	MXSPD	[1.4, 15.5]	knot
	MAXT	[28.4, 104.7]	°F
	MINT	[17.2, 83.8]	°F
	PRCP	[0, 4.11]	inch
	DT	[0, 1, 2]	/
	ST	[0.2, 0.4, 0.6, 0.8]	/
Complexity features	FD	[1.663325651, 1.917850744]	/
	HE	[0.576017672, 0.944993685]	/
	SE	[0.338285442, 1.170284745]	/
	MLE	[-0.020356277, 0.031809018]	/

Table S2 Prediction errors of multiple comparison models with different feature sets.

Feature set	Metrics	Model								
		ELM	LSTM	GRU	CEEMDAN-ELM	CEEMDAN-LSTM	CEEMDAN-GRU	VMD-ELM	VMD-LSTM	VMD-GRU
All	RMSE	2231.580154	3159.452303	2555.070743	1583.668106	1393.303049	1193.134955	1214.822922	749.224005	674.488371
	MAE	1660.826873	1960.032948	1818.081608	1256.2847984	1038.722998	891.007251	935.645852	537.922606	512.518293
	MAPE	23.654919	22.022449	24.038476	18.484645	14.628136	12.822914	13.211603	6.801322	7.095566
	R^2	0.817128	0.633439	0.760267	0.908223	0.928954	0.947921	0.942635	0.978180	0.982316
Complexity analysis only	RMSE	2186.462399	2983.894393	2440.561613	1551.270955	1371.030015	1169.055640	1178.344578	670.269160	546.501682
	MAE	1801.723449	2085.647355	1581.548358	1197.082882	991.371272	808.864458	893.175199	455.609225	389.877773
	MAPE	27.373656	26.279819	19.523384	18.285638	13.383264	10.752338	12.739047	5.465971	4.824470
	R^2	0.824448	0.673044	0.781273	0.911962	0.931186	0.950007	0.946028	0.982537	0.988391
Meteorological factor only	RMSE	2098.429106	2370.589678	2439.351515	1460.386274	1339.397250	1187.085106	1127.720886	642.514465	549.066995
	MAE	1609.558494	1680.501419	1874.099713	1128.907839	1027.005398	878.243612	871.850776	521.062853	411.110791
	MAPE	24.532402	20.788868	25.513455	16.776696	14.753368	12.576810	13.254639	7.141370	5.549112
	R^2	0.838299	0.793636	0.781490	0.921965	0.934432	0.948406	0.950566	0.983953	0.988281

XGBoost screened	RMSE	2086.331549	2716.918223	2300.972869	1418.953999	1191.727077	1134.998723	1005.942378	577.876172	447.914292
	MAE	1602.132318	2002.254127	1651.114807	1088.172136	861.348212	814.350885	717.038583	398.331519	321.934698
	MAPE	23.151721	27.155891	22.057922	15.811080	11.542723	11.044376	9.808534	5.202687	4.200876
	R^2	0.840159	0.728934	0.805578	0.926330	0.948037	0.952919	0.960666	0.987019	0.992201

Table S3 Results of evaluation of one-step, three-step and six-step forecasting for multiple comparison models with different feature sets.

Feature set	Model	1-step				3-step				6-step			
		RMSE	MAE	MAPE	R^2	RMSE	MAE	MAPE	R^2	RMSE	MAE	MAPE	R^2
All	ELM	2231.580154	1660.826873	23.654919	0.817128	2641.35692	2005.242316	28.919158	0.743802	2915.133734	2224.161997	31.557551	0.687939
	LSTM	3159.452303	1960.032948	22.022449	0.633439	3984.659081	2505.693143	27.790835	0.416952	4474.815903	2752.546443	31.113304	0.264686
	GRU	2555.070743	1818.081608	24.038476	0.760267	3217.740672	2262.747437	28.714571	0.619789	3952.779911	2625.879600	32.048062	0.426244
	CEEMDAN-ELM	1583.668106	1256.284798	18.484645	0.908223	1893.679877	1474.361226	20.891795	0.869639	2170.374788	1727.166495	24.987652	0.830551
	CEEMDAN-LSTM	1393.303049	1038.722998	14.628136	0.928954	1456.615923	1116.908931	15.717224	0.922869	1854.826487	1378.812124	19.356991	0.876341
	CEEMDAN-GRU	1193.134955	891.007251	12.822914	0.947921	1460.006041	1063.903013	14.799071	0.922589	1906.711169	1366.760538	18.881809	0.869560
	VMD-ELM	1214.822922	935.645852	13.211603	0.942635	1416.281198	1088.371919	15.083239	0.922104	1713.488089	1320.018007	18.238596	0.885477
	VMD-LSTM	749.224005	537.922606	6.801322	0.97818	1022.674376	718.759403	8.994197	0.959385	1463.398254	1047.284843	13.253124	0.916468
	VMD-GRU	**674.488371**	**512.518293**	**7.095566**	**0.982316**	823.464535	595.461851	7.888771	0.973667	1123.937807	779.970061	9.771246	0.950726
Complexity analysis only	ELM	2186.462399	1801.723449	27.373656	0.824448	2565.955905	2079.063314	30.294471	0.758219	3066.216615	2361.483769	37.140939	0.654755
	LSTM	2983.894	2085.647	26.2798	0.6730	4087.831	2696.132	31.8500	0.3863	4918.432	2965.403	34.0682	0.1116

		393	355	19	44	677	151	79	68	626	691	05	67
	GRU	2440.561613	1581.548358	19.523384	0.781273	3210.441077	1947.343787	22.949913	0.621512	3972.046824	2397.413122	27.433438	0.420637
	CEEMDAN-ELM	1551.270955	1197.082882	18.285638	0.911962	1712.017208	1313.028757	19.521182	0.893563	1994.036001	1516.030539	22.459966	0.857386
	CEEMDAN-LSTM	1371.030015	991.371272	13.383264	0.931186	1662.195331	1280.162247	18.610057	0.899473	2242.741666	1726.823837	24.801392	0.818936
	CEEMDAN-GRU	1169.05564	808.864458	10.752338	0.950007	1448.307130	1032.810263	13.845221	0.923849	1882.443665	1335.855062	18.389221	0.872923
	VMD-ELM	1178.344578	893.175199	12.739047	0.946028	1257.070137	986.738777	14.574992	0.938633	1520.626391	1208.855636	17.863104	0.909806
	VMD-LSTM	670.26916	455.609225	5.465971	0.982537	895.664177	644.946909	8.012553	0.968847	1259.981895	927.410686	11.696157	0.938076
	VMD-GRU	546.501682	389.877773	4.82447	0.988391	733.757704	535.706001	6.680359	0.979092	1030.898633	760.118319	9.644862	0.958546
Meteorological factor only	ELM	2098.429106	1609.558494	24.532402	0.838299	2636.337142	2015.615510	29.453954	0.744774	2992.962233	2330.305524	34.610078	0.671054
	LSTM	2370.589678	1680.501419	20.788868	0.793636	3193.651245	2149.787453	24.950061	0.625461	3464.445292	2333.909160	27.144352	0.559253
	GRU	2439.351515	1874.099713	25.513455	0.78149	3059.216967	2307.804635	30.259608	0.656329	3421.609749	2561.612445	33.848769	0.570084
	CEEMDAN-ELM	1460.386274	1128.907839	16.776696	0.921965	1699.312775	1304.238872	19.305724	0.895098	2053.084708	1576.970278	23.336618	0.848721
	CEEMDAN-	1339.39725	1027.005398	14.753368	0.934432	1543.904916	1186.688981	17.456439	0.913721	1906.797666	1465.091599	21.468322	0.870535

	LSTM												
	CEEMDAN-GRU	1187.085106	878.243612	12.57681	0.948406	1472.440437	1080.634220	15.818721	0.921066	1866.576201	1344.678561	19.226301	0.874313
	VMD-ELM	1127.720886	871.850776	13.254639	0.950566	1255.544198	969.110741	14.503659	0.938782	1599.842300	1262.817266	19.132594	0.900165
	VMD-LSTM	642.514465	521.062853	7.14137	0.983953	845.580409	663.252013	9.044656	0.972233	1140.246595	857.537006	11.401935	0.949286
	VMD-GRU	549.066995	411.110791	5.549112	0.988281	710.965606	517.031190	6.673557	0.980370	1028.427937	743.216449	9.511733	0.958745
XGBoost screened	ELM	2086.331549	1602.132318	23.151721	0.840159	2649.964831	2083.270678	30.556293	0.742129	3168.349846	2473.098357	36.287066	0.631372
	LSTM	2716.918223	2002.254127	27.155891	0.728934	4069.872869	2687.796031	34.941876	0.391748	4535.621048	2889.310041	33.389617	0.244567
	GRU	2300.972869	1651.114807	22.057922	0.805578	3104.304839	2085.682997	25.738308	0.646124	3947.210300	2596.630553	30.099249	0.427859
	CEEMDAN-ELM	1418.953999	1088.172136	15.81108	0.92633	1710.777989	1290.090342	18.728888	0.893678	2143.768827	1659.595441	26.295546	0.835056
	CEEMDAN-LSTM	1191.727077	861.348212	11.542723	0.948037	1426.567873	1027.565769	14.640389	0.926087	1647.061007	1194.380945	17.079924	0.902707
	CEEMDAN-GRU	1134.998723	814.350885	11.044376	0.952919	1354.109103	1006.552260	13.817427	0.933612	1741.002922	1276.307530	17.916016	0.891891
	VMD-ELM	1005.942378	717.038583	9.808534	0.960666	1212.854401	868.154072	11.898813	0.942874	1652.571524	1219.613088	16.929801	0.893475
	VMD-LSTM	577.876172	398.331519	5.202687	0.987019	777.108675	518.286729	6.455729	0.976548	1184.026919	820.333395	9.989266	0.945317

	VMD-GRU	447.914292	321.934698	4.200876	0.992201	599.747286	414.108263	5.204439	0.986031	905.755273	630.943584	7.787919	0.967999

S2. Comparative discussion with recent time series models

To evaluate the performance and competitiveness of the proposed XGBoost-VMD-GRU model incorporating data complexity features, comparative experiments were conducted with three representative and advanced time series forecasting models: Transformer[1], Informer[2], and TimesNet [3]. This section evaluates the forecasting performance using RMSE, MAE, MAPE, and R^2, under a unified data preprocessing procedure and identical training-testing splits for all models. The experiments are conducted based on four input feature schemes, as listed in Table 5: All features, Only complexity-related features, Only meteorological features, and Features selected by XGBoost. The summarized results are shown in Table S4, and the corresponding prediction curves are presented in Figure S1.

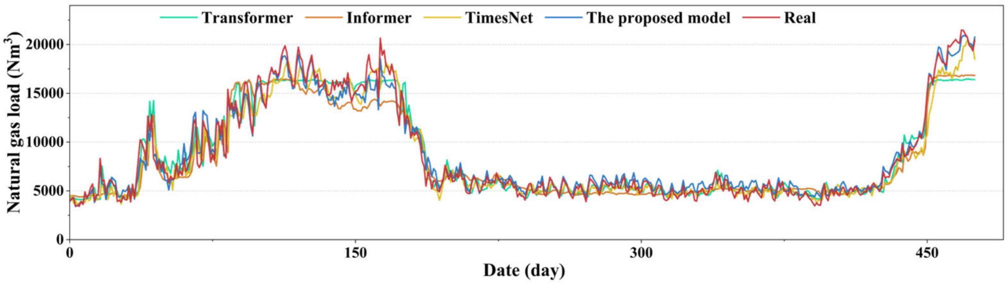

(a) A complete set of unfiltered features.

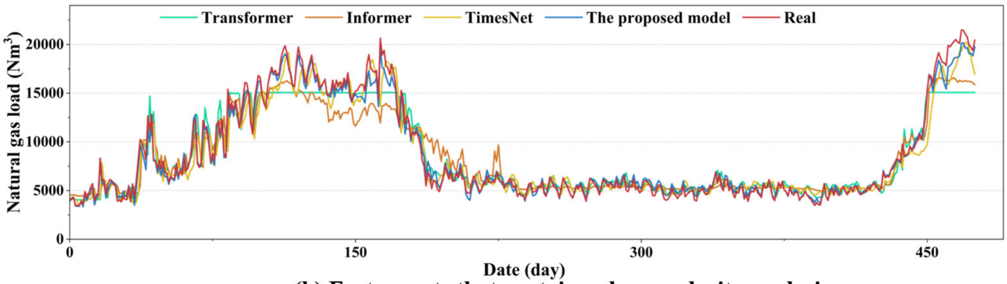

(b) Feature sets that contain only complexity analysis.

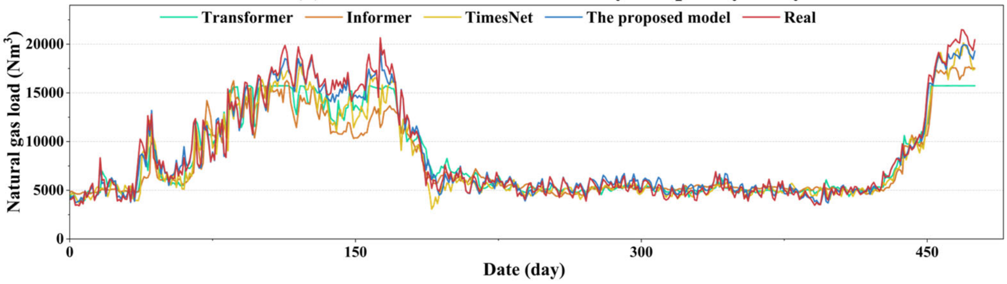

(c)Feature sets containing only weather, date type, and season type.

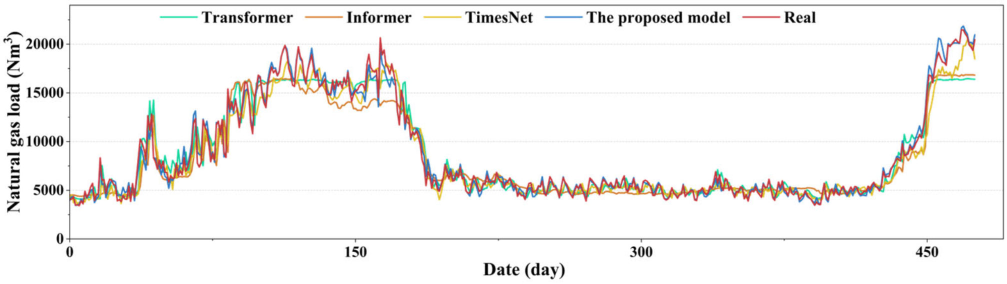

(d)Key feature sets selected by XGBoost.

Figure S1 Comparison of prediction results between Transformer, Informer, TimesNet and the proposed model.

This section illustrates that the XGBoost-VMD-GRU model consistently achieves the highest prediction accuracy across all feature configurations in daily natural gas load forecasting. Notably, it maintains stable performance during peak-valley periods characterized by high volatility and nonlinearity. TimesNet demonstrates the second-best performance. Its 2D convolution-based architecture enables effective extraction of multi-scale periodic patterns, leading to reliable predictions. Compared to the proposed method, Transformer and Informer exhibit relatively weaker performance, particularly in capturing peak load fluctuations. This may be attributed to the tendency of their self-attention mechanisms to dilute important information when modeling short-term critical points. As shown in Figure S1(d), incorporating screened complexity and meteorological features leads to predicted load curves that are more closely aligned with the actual values. While this study does not adopt the most advanced model architecture, it incorporates complexity analysis, structured feature engineering, and mode decomposition, demonstrating strong adaptability in modeling load data characterized by periodic patterns, structured disturbances, and nonlinear dynamics.

Table S4 Comparison of prediction performance of Transformer, Informer, TimesNet, and the proposed model across different feature sets.

Feature set	Model	RMSE	MAE	MAPE	R^2
All	Transformer	2118.3003	1468.1978	16.2804	0.8449
	Informer	2398.4419	1633.3024	16.9172	0.8015
	TimesNet	1822.5176	1263.4180	13.7671	0.8859
	The proposed model	674.488371	512.518293	7.095566	0.982316
Complexity analysis only	Transformer	2035.5245	1360.6453	15.1069	0.8568
	Informer	2159.5303	1498.6805	17.4852	0.8391
	TimesNet	1610.3688	1113.6260	13.2559	0.9109
	The proposed model	546.501682	389.877773	4.82447	0.988391
Meteorological factor only	Transformer	2019.7211	1386.7389	15.4102	0.8590
	Informer	2332.4355	1576.7061	16.9145	0.8123
	TimesNet	1757.2935	1218.6467	13.7246	0.8939
	The proposed model	549.066995	411.110791	5.549112	0.988281
XGBoost screened	Transformer	1713.9998	1171.9033	13.6650	0.8984
	Informer	1893.3591	1302.1177	14.7988	0.8763
	TimesNet	1516.3493	1064.2646	12.2763	0.9210
	The proposed model	447.914292	321.934698	4.200876	0.992201

Table S4 further demonstrates that under the "XGBoost screened" feature configuration, all models exhibit significantly lower RMSE, MAE, and MAPE values compared to other feature sets. This indicates that integrating data complexity features and key meteorological variables via targeted feature engineering improves the robustness of forecasting models in non-stationary settings. Under the selected feature

set, RMSE decreased to 447.9143 and R^2 improved to 0.9922, outperforming TimesNet (RMSE: 1516.3493, R^2: 0.9210), Transformer (RMSE: 1713.9998, R^2: 0.8984), and Informer (RMSE: 1893.3591, R^2: 0.8763).

In summary, this study demonstrates that the hybrid forecasting framework integrating mode decomposition and complexity-based feature modeling offers clear advantages when applied to energy load data characterized by strong nonlinearity and periodicity. Future research could explore the integration of complexity analysis, mode decomposition techniques, and advanced time series models to further enhance predictive accuracy and model robustness.

SI References

[1] Vaswani A, Shazeer N, Parmar N, Uszkoreit J, Jones L, Gomez AN, Kaiser Ł, Polosukhin I. Attention is all you need. Advances in neural information processing systems. 2017;30.http://arxiv.org/abs/1706.03762.

[2] Zhou H, Zhang S, Peng J, Zhang S, Li J, Xiong H, Zhang W. Informer: Beyond efficient transformer for long sequence time-series forecasting. InProceedings of the AAAI conference on artificial intelligence 2021; 35(12): 11106-11115. https://doi.org/10.1609/aaai.v35i12.17325.

[3] Wu H, Hu T, Liu Y, Zhou H, Wang J, Long M. Timesnet: Temporal 2d-variation modeling for general time series analysis. arxiv preprint arxiv:2210.02186. 2022. https://doi.org/10.48550/arXiv.2210.02186.
